# Mckusick-Kaufman Syndrome: Diagnosis and Management

**Published:** 2014-01-01

**Authors:** Ahmed H. Al-Salem, Sharif H. Abdel-Aziz

**Affiliations:** Department of Pediatric Surgery, Maternity and Children Hospital, Dammam, Saudi Arabia

**Dear Sir**

A female newborn, a product of consanguineous marriage born by caesarian section delivery with a birth weight of 3.75 kg, was admitted to our hospital with abdominal distension. Antenatal ultrasound showed congenital heart disease and a pelvic mass. On examination, she had lower abdominal mass emanating from the pelvis. There was polydactly and syndactly of the right hand and polydactly of the right foot. There was also an anterior ectopic anus. Cardiovascular work up revealed common atrio-ventricular (A-V) canal with single atrium, moderate patent ductus arteriosus and severe A-V regurgitation. Abdominal ultrasound and CT-scan showed hydrometrocolpos with bilateral hydronephrosis and left perinephric fluid collection (Fig. 1). Surgery revealed hydrometrocolpos secondary to a low vaginal atresia. An abdomino-perineal vaginal pull through was performed. Post-operative period was uneventful.

**Figure:**
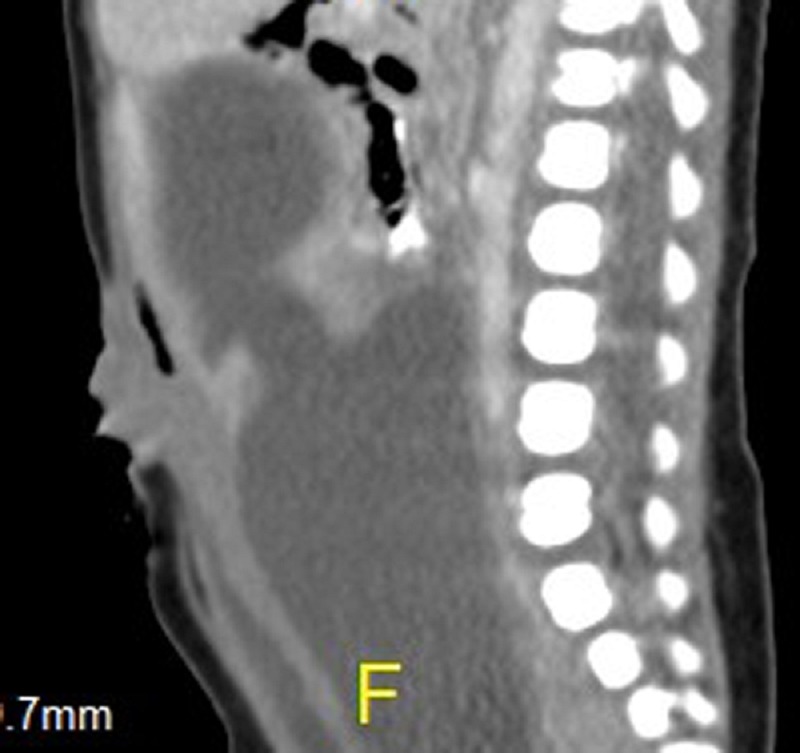
Figure 1: CT-Scan showing hydrometrocolpos. Note the dilated vagina and uterus.

McKusick-Kaufman syndrome is characterized by the triad of hydrometrocolpos, postaxial polydactyly and congenital heart disease (1-4). This condition was first described in the Amish population [1]. Hydrometrocolpos is present in 80–95% of females; postaxial polydactyly is present in 90% of patients; and congenital heart diseases have been described in 15%-20% of patients with McKusick-Kaufman syndrome.

Clinically, the patient may present with a large, cystic abdominal mass which can be sufficiently large to cause intestinal obstruction, urinary outflow obstruction leading to hydroureter and hydronephrosis, and/or elevation of the diaphragm resulting in breathing difficulties, associated with postaxial polydactyly and congenital heart defects. The index case had polydactyly of the right hand and right foot; hydrometrocolpos secondary to low vaginal atresia; and common atrio-ventricular (A-V) canal with single atrium, moderate patent ductus arteriosus and severe A-V regurgitation. Other cardiac malformations include ventricular septal defect, atrial septal defect, small aorta and hypoplastic left ventricle, tetrology of Fallot, and patent ductus arteriosus. The severity of the associated congenital heart malformations determines the final outcome. Cardiac evaluation and echocardiogram should form part of the preoperative investigations of these patients to accurately define the associated cardiovascular malformations. Additional less commonly associated abnormalities include imperforate anus, rectovaginal or vesicovaginal fistulae, Hirschsprung’s disease, anterior ectopic anus and malrotation [1-3]. Our patient had also an associated anterior ectopic anus.

It is important to define anatomic abnormality leading to the hydrometrocolpos. We found CT-scan valuable in this regard. MRI has been reported to be more valuable than ultrasound and CT-scan in delineating the vaginal anatomical defect and the hydroureter and hydronephrosis [2]. Genitography is an unnecessary invasive investigation that may be harmful leading to secondary infection with subsequent pyometrocolpos. Pyometrocolpos which is a serious complication calls for early and rapid evaluation and treatment of these patients. Hydrometrocolpos secondary to vaginal atresia is treated with abdomino-perineal vaginal pull through as we did in our case.

## Footnotes

**Source of Support:** Nil

**Conflict of Interest:** None

